# 1893. Mathematical modeling the impact of the age-dependent latent tuberculosis treatment on active tuberculosis in South Korea

**DOI:** 10.1093/ofid/ofad500.1721

**Published:** 2023-11-27

**Authors:** Hye Seong, Yunjeong Lee, Jiyeon Seo, Hakjun Hyun, Jin Gu Yoon, Eliel Nham, Ji Yun Noh, Hee Jin Cheong, Woo Joo Kim, Jeehyun Lee, Joon Young Song

**Affiliations:** Division of Infectious Diseases, Department of Internal Medicine, Korea University College of Medicine, Seoul, South Korea, Seoul, Seoul-t'ukpyolsi, Republic of Korea; School of Mathematics and Computing (Computational Science and Engineering), Yonsei University, Seoul, South Korea, Seoul, Seoul-t'ukpyolsi, Republic of Korea; School of Mathematics and Computing (Computational Science and Engineering), Yonsei University, Seoul, South Korea, Seoul, Seoul-t'ukpyolsi, Republic of Korea; Korea University Guro Hospital, Seoul, Seoul-t'ukpyolsi, Republic of Korea; Division of Infectious Diseases, Department of Internal Medicine, Korea University College of Medicine, Seoul, South Korea, Seoul, Seoul-t'ukpyolsi, Republic of Korea; Division of Infectious Diseases, Department of Internal Medicine, Korea University College of Medicine, Seoul, South Korea, Seoul, Seoul-t'ukpyolsi, Republic of Korea; Division of Infectious Diseases, Department of Internal Medicine, Korea University College of Medicine, Seoul, South Korea, Seoul, Seoul-t'ukpyolsi, Republic of Korea; Division of Infectious Diseases, Department of Internal Medicine, Korea University College of Medicine, Seoul, South Korea, Seoul, Seoul-t'ukpyolsi, Republic of Korea; Division of Infectious Diseases, Department of Internal Medicine, Korea University College of Medicine, Seoul, South Korea, Seoul, Seoul-t'ukpyolsi, Republic of Korea; Yonsei University, Seodaemun-gu, Seoul-t'ukpyolsi, Republic of Korea; Division of Infectious Diseases, Department of Internal Medicine, Korea University College of Medicine, Seoul, South Korea, Seoul, Seoul-t'ukpyolsi, Republic of Korea

## Abstract

**Background:**

As most of the tuberculosis (TB) cases occur from latent TB (LTBI) reactivation, effective therapeutic interventions for LTBI are crucial to national TB control. Age is one of the important factors affecting LTBI treatment and management. This study assessed the impact of the age-dependent intervention on the treatment rate, the probability of treatment success for LTBI, and the overall incidence of active TB cases in South Korea.

**Methods:**

We developed an age-structured dynamic compartmental model to describe the person-to-person transmission of TB focused on latent TB in South Korea. We calibrated the model to an annual number of active TB cases from 2011 to 2018 reported by Korea Disease Control and Prevention Agency. The model estimated averted active TB cases for 30 years, and sensitivity analysis was performed by changing the treatment coverage and success rates for LTBI, stratified by age groups.

The mathematical model for tuberculosis transmission in South Korea

**Figure:**
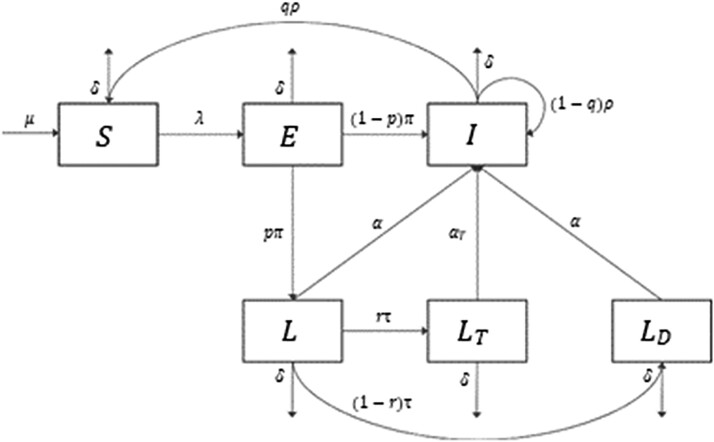

**Results:**

In the model simulation, the number of new active TB cases gradually decreased in 19-34 and 35-64 age groups but gradually increased in the elderly. Among three age groups, the intervention of LTBI in 35-64 age group markedly reduced total active TB cases by reducing both acute infection and reactivation from LTBI cases. When the LTBI treatment rate increased by 4-times in 35-64 age group, the 32,785 active TB cases were averted, while in 19-34 and ≥65 age groups, 11,597 and 5,678 cases were averted, respectively. When changing the probability of treatment success, similar results were shown also. The 19-35 age group was most affected by the change in the LTBI treatment success rate compared to other age groups. Assuming the probability of treatment success approached 100%, 1,290, 3,208 and 822 active TB cases were averted in 19-34, 35-64 and ≥65 age groups, respectively.

**Figure:**
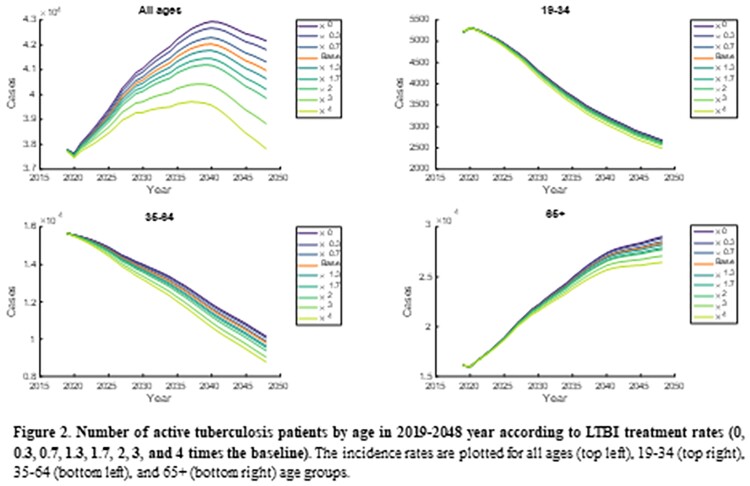

The incidence rates are plotted for all ages (top left), 19-34 (top right), 35-64 (bottom left), and 65+ (bottom right) age groups.

**Figure:**
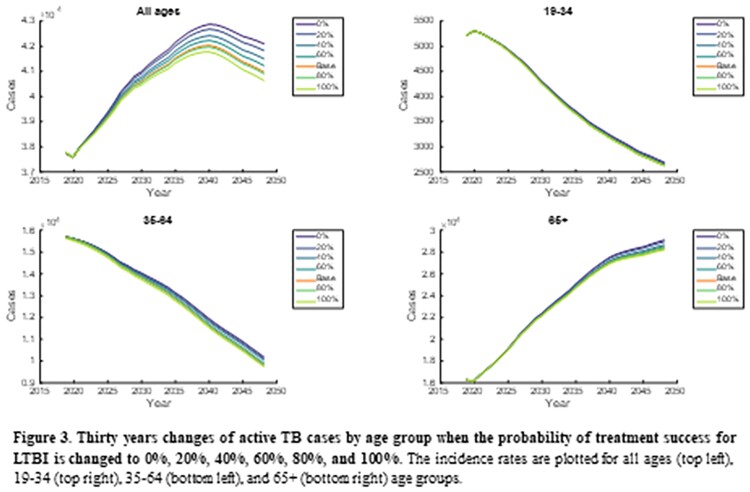

The incidence rates are plotted for all ages (top left), 19-34 (top right), 35-64 (bottom left), and 65+ (bottom right) age groups.

Active TB cases in 2048 year according to changes of LTBI treatment rate for each age group

**Figure:**
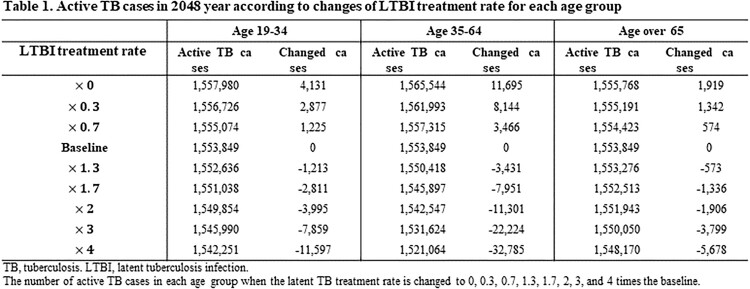

**Conclusion:**

The model simulation suggested that changes in the treatment coverage and success rates of LTBI may have different effects by the age group. LTBI control measures affected the 35-64 year age group more significantly than the other age groups.

**Figure:**
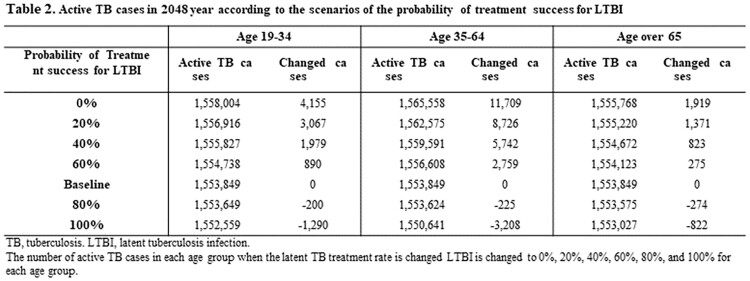

**Disclosures:**

**Hee Jin Cheong, M.D.,Ph.D.**, Sequiris: Advisor/Consultant

